# Phytoconstituents and Nutritional Properties of the Fruits of* Eleutherococcus divaricatus* and* Eleutherococcus sessiliflorus*: A Study of Non-European Species Cultivated in Poland

**DOI:** 10.1155/2017/8374295

**Published:** 2017-01-15

**Authors:** Daniel Załuski, Marta Olech, Robert Verpoorte, Inamullah Khan, Rafał Kuźniewski, Renata Nowak

**Affiliations:** ^1^Department of Pharmacognosy, Ludwik Rydygier Collegium Medicum, Nicolaus Copernicus University, 9 Marie Curie-Skłodowska Street, 85-094 Bydgoszcz, Poland; ^2^Chair and Department of Pharmaceutical Botany, Medical University of Lublin, 1 Chodźki Street, 20-093 Lublin, Poland; ^3^Natural Products Laboratory, Institute of Biology, Leiden University, 2300 RA Leiden, Netherlands; ^4^Department of Pharmacy, University of Peshawar, Peshawar 25120, Pakistan

## Abstract

*Eleutherococcus* fruits have been consumed in Russia and Asia throughout the centuries. Currently, there is an increasing interest in these products by the community of Western Europe. Many people suffer from micronutrient deficiencies, known as malnutrition, which consequently influences body condition. The aim of this study was to investigate pharmaconutrition, proximate, mineral, and fatty acid composition, total phenolics content, and total flavonoids content of* Eleutherococcus divaricatus* and* Eleutherococcus sessiliflorus* fruits cultivated in Poland.* Eleutherococcus divaricatus* and* E. sessiliflorus* contain a high amount of protein and fibres (16.70% and 12.28%; 61.41% and 45.63%, resp.). The fruits were generally high in K (21 g/kg) and low in sodium (0.001 g/kg). In terms of fatty acid composition, both species had a high amount of monounsaturated fatty acids (54.84–57.95%) and polyunsaturated fatty acids (36.22–37.0%). Using LC-ESI-MS/MS, protocatechuic acid has been identified as the most abundant compound, ranging from 260 to 810 *μ*g/100 g DE. Among flavonoids, hyperoside was found to be in the highest amount (120–780 *μ*g/100 g DE). Considering a rich chemical composition of the fruits, a better understanding of their health benefits is important in order to increase their utility and to enrich dietary sources of health promoting compounds. Because of a high amount of protein and a low calorific value, the fruits should be considered food for vegans or vegetarians.

## 1. Introduction

Species from the* Eleutherococcus* Maxim. genus are a valuable source of eleutherosides, phenolic acids, flavonoids, anthocyanins, triterpenoids, and biopolymers. One of the most known species of that genus is* E. senticosus*, which is very popular as a dietary supplement in Asia and the United States. Its products are used in the form of capsule, powder, and teabag, as health foods and drugs [1, 2]. The main compounds, including eleutherosides, phenolic acids, and flavonoids, were characterised by HPLC, HPTLC, and LC-MS methods [3]. Apart from the aforementioned compounds, these species contain oleanolic and betulinic acids, chiisanoside, lipid acids, essential oil, and sesamin [4, 5].

Several reports have revealed that* E. divaricatus* and* E. sessiliflorus* fruits are beneficial for human health, and currently there has been a growing research interest with regard to the products of these fruits used for consumption. According to Załuski's previous studies, the fruits of species cultivated in Poland act as antioxidants, induce apoptosis in Jurkat 45 leukemic cell line, and inhibit the activity of MMP-1, MMP-2, MMP-3, and MMP-9. It is thought that chiisanoside, which was identified in the leaves, decreases the absorption of lipids. The roots and stems of* E. sessiliflorus* have been shown to have antipancreatic lipase and anti-inflammatory activities. With regard to its pharmacological aspects,* E. sessiliflorus* has been used in traditional medical protocols as a tonic, analgesic, antihypertensive, and antidiabetic agent. Załuski's previous studies have shown that the fruits may reduce DPPH^*∗*^ radical, inhibit lipid peroxidation, and have an ion-chelating ability [6–10]. In food industry, only species native to Asia are used, while species cultivated in Europe are not yet used commercially. It is important that tested species are cultivated in other geographical zones (Poland) than native ones (Asia). The Polish climate conditions may have an influence on the chemical profile and nutritional value of fruits. They were successfully cultivated at the botanical garden in Rogów, which lies in the Central Polish Lowlands region with geographic data 51°49′N and 19°53′E. The vegetative period lasts for 212 days and the average annual precipitation is 596 mm, of which 80% occurs during the vegetative period. The average annual air temperature is 7.2°C. The average long-term temperature is −20.1°C, which classified the garden to the 6bth subclimate (according to “USDA Frost Hardiness Zones”) and to the second zone according to Kórnik's category. These plants are grown on the acidic, luvic, and sandy soils [11].

Phenolic compounds, known as nonnutritional ingredients in food, constitute one of the most widely occurring groups of phytochemicals with a wide range of physiological properties [12]. They are components of many fruits and vegetables, which are associated with health benefits after their consumption [13]. Clinical trials and epidemiological studies have established that dietary intake of fruits is strongly associated with a reduced risk of the civilization diseases. In the human body, they act as antiallergenic, antiatherogenic, anti-inflammatory, antimicrobial, antioxidant, and antithrombotic agents [12, 14].

A study of the pharmaconutrients panel of fruits can give us a better understanding of their potential beneficial effects on human health. It is a common opinion that the origin of most illnesses is due to inappropriate nutrition. Keeping in mind their long-term use by the Asians, we have decided to evaluate the quality of* E. divaricatus* and* E. sessiliflorus* cultivated in Polish climate conditions as a pharmaconutrient material and potential dietary and/or pharmaceutical product. Because those species are cultivated in another geographical zone than a native one, it is necessary to study the composition of the raw material, to ensure product quality. No comprehensive data have been reported on the proximate, mineral, fatty acids, phenolics, and flavonoids composition and caloric value of the fruits. Apart from the raw extracts, we investigated also the infusions, as very popular homemade beverages, in many cases containing different fruits.

## 2. Experimental

### 2.1. Plant Material

The fruits of* E. divaricatus* (Siebold et Zucc.) S. Y. Hu and* E. sessiliflorus* (Rupr. & Maxim.) S. Y. Hu were collected at the arboretum in Rogów (Poland) in October 2016. All plant samples were deposited at the Department of Pharmacognosy, Collegium Medicum, Bydgoszcz, Poland (Cat. number ED 01-2016; ES 02-2016).

### 2.2. Chemicals

Folin-Ciocalteau reagent, DMSO, gallic acid, quercetin, and hesperetin were purchased from ChromaDex. Standards of gallic, protocatechuic, gentisic, 4-OH-benzoic, vanillic, caffeic, syringic, p-coumaric, ferulic, salicylic, veratric, sinapic, 3-OH-cinnamic, and rosmarinic acid, luteolin 7-glucoside, luteolin 3,7-diglucoside, rutin, hyperoside, isoquercetin, naringin, naringenin 7-glucoside, quercitrin, apigenin 7-glucoside, and LC grade acetonitrile were purchased from Sigma-Aldrich Fine Chemicals (St. Louis, MO, USA). Kaempferol 3-rutinoside and astragalin were from Carl Roth (Karlsruhe, Germany). Luteolin 4′-O-glucoside was obtained from LGC Standards (Dziekanów Leśny, Poland). 2,4-DNPH, ethanol, FeCl_3_, and HNO_3_ were obtained from POCH (Lublin, Poland). LC grade methanol (MeOH) was purchased from J. T. Baker (Phillipsburg, USA). LC grade water was prepared using a Millipore Direct-Q3 purification system (Bedford, MA, USA). All others reagents were of analytical grade.

### 2.3. Accelerated Solvent Extraction (ASE)

The air-dried and powdered fruits (5 g each) were placed in an extraction cell with 30 g of neutral silica gel. The ASE cell was placed into ASE for the extraction process (Dionex system). During the extraction process, 75% ethanol was delivered into the extraction cell. Pressure (1000 psi) was applied to maintain the solvent in its liquid state. The extraction process was repeated three times using 10 mL ethanol. The extraction temperature was 40°C, and the extraction time was 15 min. Following extraction, the extract containing the target analytes was purged from the cell using nitrogen into a collection vial for analysis. After the three extraction cycles, 30 mL of extracts was obtained. The solvents were dried with an evaporator under vacuum conditions at 45°C and subjected to lyophilisation.

### 2.4. Infusion Preparation

The infusion was prepared by adding 50 mL of distilled water (95°C) to 5 g of fruits. The infusions were brewed for 15 minutes and were then filtered over Whatman No. 1 paper. The aqueous extracts were frozen and lyophilised.

### 2.5. Proximate Composition

The fruits were analysed for proximate composition: moisture by air drying at 105°C for 2 h, total fat by extraction with hexane in Soxhlet's apparatus, protein by Kjeldahl's method, and ash by direct analysis at 550°C for 6 h. Total carbohydrates were calculated by difference. Values represent averages of triplicate determinations performed for all analyses.

### 2.6. AAS of Minerals

0.5 g of the dried and ground fruits was put into a burning cup, and 2 mL of pure HNO_3_ was added. The samples were incinerated in a MARS 5 microwave oven (Manufactured by CEM Corporation, USA) at a temperature of 90°C for 15 min and next at 120°C for 10 min and 210°C for 30 min, and the solution was diluted to 100 mL with water. Minerals content was determined with a Varian SpektrAA 280FS + Autosampler SPS 3 spectrometer. Minerals and trace elements were determined using the instrumental conditions recommended for each mineral and were calculated based on the respective standard curve.

### 2.7. GC-FID Fatty Acid Analysis

Fatty acids were extracted with hexane in Soxhlet's apparatus. A highly sensitive and accurate multiplex gas chromatography-linear ion trap technique was used to identify components of lipid fraction. GC/MS/MS was performed using Varian 4000 GC/MS/MS chromatograph. The GC conditions were as follows: VF-5 ms fused silica capillary column (30 × 0.32 mm, film thickness: 0.25 *μ*m), with the oven temperature programmed at a rate of 3°C from 200 (held for 10 min) to 240°C (held for 4.67 min) and injector kept at 250°C and detector at 300°C, with split ratio 1 : 50. Helium was used as the carrier gas with a constant flow rate of 2.5 mL/min. The sample size was 1 *μ*L in hexane. Compounds were identified using Galaxie™ Chromatography Data System. The following acids were analysed: C6:0 caproic; C8:0 caprylic; C10:0 capric; C11:0 undecanoic; C12:0 lauric; C13:0 tridecanoic; C14:0 myristic; C14:1 myristoleic; C15:0 pentadecanoic; C15:1 cis-10-pentadecenoic; C16:0 palmitic; C16:1 palmitoleic; C17:0 heptadecanoic; C17:1 cis-10-heptadecenoic; C18:0 stearic; cis-C18:1n–9 oleic; trans-C18:1n–9 elaidic; cis-C18:2n–6 linoleic; trans-C18:2n–6 linolelaidic; C18:3n–6 *γ*-linolenic; C18:3n–3 *α*-linolenic; C20:0 arachidic; C20:1 cis-5-eicosenoic; C20:2 cis-11,14-eicosadienoic; C20:3n–6 cis-8,11,14-eicosatrienoic; C20:4n–6 arachidonic; C20:5n–3 eicosapentaenoic; C21:0 heneicosanoic; C22:0 behenic; C22:1n–9 erucic; C22:2 cis-13,16-docosadienoic; C23:0 tricosanoic; C24:0 lignoceric; C24:1n–9 nervonic. The data of total lipids were statistically analysed and expressed as mean ± standard deviation.

### 2.8. Total Phenolic Content (TPC)

The total phenolic content of extracts was determined using the method of Singleton and Rossi [15]. TPC was expressed as gallic acid equivalents (20–100 *μ*g/mL; *y* = 0.0026*x* + 0.044; *r*^2^ = 0.999; GAE/g dry extract). The experiments were done in triplicate.

### 2.9. Total Flavonoid Content (TFC)

The TFC in investigated samples was determined using aluminium chloride and 2,4-dinitrophenylhydrazine colorimetric methods [16]. TFC were expressed as means (±SE) mg of quercetin equivalent (20–100 *μ*g/mL; *y* = 0.0041*x* + 0.236; *r*^2^ = 0.999; QEs/g dry extract for FeCl_3_ method) and as means (±SE) mg of hesperetin equivalent (HEs/g dry extract for DNPH method; 250–1000 *μ*g/mL; *y* = 6.374*x* − 0.098; *r*^2^ = 0.988). The experiments were done in triplicate.

### 2.10. LC-ESI-MS/MS Conditions of Analysis of Phenolic Acids and Flavonoids

To evaluate phenolic acids content, the samples were analysed using a modified LC-ESI-MS/MS version of Nowacka et al. [17], with the levels of flavonoid glycosides as reported below. An Agilent 1200 Series HPLC system (Agilent Technologies, USA) equipped with a binary gradient solvent pump, a degasser, an autosampler, and column oven connected to 3200 QTRAP mass spectrometer (AB Sciex, USA) was used. Chromatographic separation was carried out at 25°C, on an Eclipse XDB-C18 column (4.6 × 150 mm, 5 *μ*m particle size; Agilent Technologies, USA) with a mobile phase consisting of water containing 0.1% HCOOH (solvent A) and acetonitrile containing 0.1% HCOOH (solvent B), using 5 *μ*L injections. The flow rate was 450 *μ*L min^−1^ and the gradient was as follows: 0-1 min, 18% B; 1.5–5.5 min, 20% B; 7–10 min, 25% B; 13–15 min, 60% B; 17–21 min, 18% B. The QTRAP-MS system was equipped with electrospray ionisation source (ESI) operated in the negative-ion mode. ESI worked at the following conditions: capillary temperature of 500°C, curtain gas at 25 psi, nebulizer gas at 50 psi, and negative-ionisation mode source voltage of −4500 V. Nitrogen was used as curtain and collision gas. For each compound, the optimum conditions of Multiple Reaction Mode (MRM) were determined in the infusion mode. The data was acquired and processed using Analyst 1.5 software (AB Sciex, USA). Triplicate injections were made for each standard solution and sample. The analytes were identified by comparing retention time and* m/z* values obtained by MS and MS^2^ with the mass spectra from corresponding standards tested under the same conditions. The calibration curves obtained in MRM mode were used for quantification of all analytes. The identified phenolic acids were quantified on the basis of their peak areas and comparison with a calibration curve obtained with the corresponding standards. Linearity ranges for calibration curves were specified. The limit of detection (LOD) and limit of quantification (LOQ) for phenolic compounds were determined at a signal-to-noise ratio of 3 : 1 and 10 : 1, respectively, by injecting a series of dilute solutions with known concentrations.

### 2.11. Statistical Analysis

All determination was performed in triplicate. The obtained data were subjected to statistical analysis using Statistica 7.0. (StatSoft, Cracow). The evaluations were analysed for one-factor variance analysis. Statistical differences between the treatment groups were estimated by Spearman's (*R*) and Pearson's (*r*) test. All statistical tests were carried out at significance level of *α* = 0.05.

## 3. Results and Discussion

### 3.1. Proximate Composition and Calorific Value

The results of proximate analysis are presented in Table 1.* Eleutherococcus divaricatus* contains high proportions of protein and fibres compared to* E. sessiliflorus*, whereas* E. sessiliflorus* has a higher level of carbohydrates. The carbohydrates content in* E. sessiliflorus* (25.7%) is quite high when compared to* E. divaricatus* (2.5%), which can be appropriate in formulating high carbohydrate diets. The ash content of 5.53 and 4.89% makes the fruits a good source of minerals for consumers.

Suárez-Martínez et al. reported on the content of protein in* Phaseolus angularis*,* P. lunatus*,* P. vulgaris*, and* P. mungo* in the range of 22.9–26.2%, respectively. Overall, the content of bean proteins is said to be at the level of 20–30%. In turn, soybean proteins represent about 35–40% on a dry weight basis. Despite the fact that the soybean is receiving increasing attention with respect to its health effects, it is also a well-recognized allergenic food for sensitive people. Additionally, soybean should not be ingested in a high amount by males because soy food and soy isoflavones are associated with lower sperm concentration [18, 19]. In this case, particular attention should be directed to the vegetarian or vegan males. Taking into account the proximate composition, it can be pointed out that the fruits of* E. divaricatus* should enrich any diet where high protein and fibre content is needed. Taking into account the calorific value, the fruits may be used in slimming diet.

### 3.2. Mineral Content

Antioxidant activity of plants is, very often, associated with the amount of mineral constituents. Some of them (Se, Zn, Mn, and Cu) are often thought to be a dietary antioxidant protecting cells from oxidative damage [20]. The concentrations of the mineral components of the fruits according to the mineralisation and identification methods are reported in Table 2. The variation in the amount of minerals between two species has been noticed. The main elements of both species were Ca and K. The fruits of* E. divaricatus* have a high level of Mn and Zn compared to* E. sessiliflorus*.* Eleutherococcus divaricatus* contains a high amount of Fe.

Other studies indicated that the* E. senticosus* fruits, collected in Korea, contain 465, 1433, 199, and 13 mg/kg dry weight of Ca, Mg, Mn, and Zn, respectively [21]. Compared with species cultivated in Poland, there is a difference in the level of Ca and Mn. Both species contain higher level of Ca and lower level of Mn. A major finding is that the species native to Asia does not contain Fe, a factor that excludes these fruits as an ingredient of antianemic diet. It is worth noting that* E. divaricatus* has higher Fe content than the* Rosa canina* L. fruits (27.0 mg/kg), which is very popular in the diet of the Europeans. Moreover, the results obtained in the present study indicated a higher content of Mn, Zn, and Cu than that in the* Rosa canina* L. and* Rosa damascena* Mill. fruits [22]. It should be mentioned that* E. divaricatus* contains more Zn and Mn than walnut of kernels (from 17.9 to 20.6 and 17.5 to 22.2 mg/kg). According to a WHO report, over 2000 million people in developing countries have iron deficiency anemia [23]. The global burden of disease estimates showed that, among the 26 major risk factors of the global burden of disease, iron deficiency ranks ninth overall, while zinc deficiency is eleventh [24]. In that case, the fruits, especially of* E. divaricatus*, should be considered a new dietary ingredient that may be included in the antianemic diet. In order to receive the best results, a combination of these fruits has to be included in the diet.

### 3.3. Fatty Acid Composition

A total of 36 different fatty acids were identified by GC-FID analysis. 16 different fatty acids were identified in* E. sessiliflorus*, and 14 were identified in* E. divaricatus*, contained at various concentrations in all the analysed fruits (Table 3). The fruits of* E. sessiliflorus* have a threefold higher amount of *α*-linolenic acid (ALA) than* E. divaricatus* (2.27 and 0.79%), the acid whose occurrence is limited. The *α*-linolenic acid is recognized as a promising therapeutic agent for numerous health disorders acting as the preventive and neuroprotective constituent of the human diet. The predominant unsaturated fatty acids obtained were oleic acid and elaidic acid up to 57%. Overall, in the present study, a majority of the fatty acids were monounsaturated (57.95% and 54,84% in* E. divaricatus* and* E. sessiliflorus*, resp.). The MUFA content was higher than that of walnut, which is an important ingredient of the European diet. Another popular product is virgin coconut oil, which is suggested as a functional food with a high amount of MUFA and PUFA. However, this oil contains a lower amount of C18:1 (6.5%) than the analysed samples. It has been established that MUFA has a beneficial effect on health by raising high-density lipoprotein cholesterol (HDLC) [25]. Apart from MUFA, the high content of PUFA and omega-6 has also been assayed.

### 3.4. TPC and TFC in the Fruits

As it was shown in Table 4, all ethanol extracts contain a higher amount of TPC than the infusions (45.3 and 52.3 mg/g DE for* E. divaricatus* and* E. sessiliflorus*, resp.). In turn, the summed amount of flavonoids ranged from 18.4 to 23.0 mg/g DE for* E. divaricatus* and* E. sessiliflorus*. We found that flavonoid content obtained by aluminium chloride reaction was much higher than those obtained by DNPH reaction. This can indicate that the species contain more compounds with the OH group which are responsible for the high antioxidant activity of the extracts.

The contents of phenolic compounds were within the range of those previously reported for the various* Eleutherococcus* species, cultivated in Poland. Załuski and Janeczko [26] showed that the fresh fruits of* E. divaricatus* and* E. sessiliflorus* contain 6.1 and 6.9 mg/g dry sample of polyphenols. Shohael et al. [27] studied* E. sessiliflorus* growing in Korea but reported a lower concentration of phenols than that now estimated. The TPC content found in the 75% ethanolic extracts from the spring leaves ranged from 20.3 to 37.2 mg/g, followed by the fresh fruits (6.1–19.7 mg/g) and the roots (6.9–10.6 mg/g). Jang et al. [28] revealed that the fruits of* E. senticosus* collected in Korea contained from 197.9 to 334.3 mg/g of polyphenols, while the TFC ranged from 41.2 to 203.7 mg/g.

Our findings showed that the investigated* Eleutherococcus* fruits contain more TPC than the blueberries fruits, which in Poland or other European countries are very widely used in food products and for medical purposes and are recognized as rich sources of polyphenols. According to Grace et al. [29], blueberries contain from 22.7 to 39.3 mg/g extract of polyphenols. According to Załuski and Janeczko [26], the content of TPC and TFC in the fruits is not changed during storage and quantified between 4.11 and 4.35 g/100 g for the freshly dried fruits from* E. senticosus* and* E. henryi*. After 1-year storage, the amount did not change significantly and was between 3.85 and 4.13 for* E. senticosus* and* E. henryi*. Heo et al. [21] reported a lower concentration of polyphenols and flavonoids in ethanol, methanol, and water extract of the* E. senticosus* fruits growing in Korea (0.3, 0.6, and 0.6% and 0.20, 0.23, and 0.3%, resp.) than that now estimated.

### 3.5. LC-ESI-MS/MS Analyses for Phenolic Acids and Flavonoids

Results of the optimization of conditions of LC-ESI-MS/MS analysis are given in Tables S1, S2, S3, and S4 in Supplementary Material available online at https://doi.org/10.1155/2017/8374295. The concentrations of individual compounds, which were quantified by comparison of peak areas with the calibration curves obtained for the corresponding standards, are reported in Table 5. Among fourteen phenolic acids (gallic, protocatechuic, gentisic, 4-OH-benzoic, 3-OH-benzoic, vanillic, syringic,* p-*coumaric, ferulic, veratric, salicylic, 3-OH-cinnamic, sinapic, and rosmarinic), just five were qualitatively and quantitatively determined in the fruits (protocatechuic, caffeic,* p-*coumaric, ferulic, and salicylic). Protocatechuic acid occurs in the highest amount (260–810 *μ*g/100 g DE). In turn, LC-ESI-MS/MS analysis of flavonoids revealed the presence of six flavonoids, of which hyperoside was determined in the highest amount (120–780 *μ*g/100 g DE). This is the first time astragalin is found in* Eleutherococcus* spp. Overall, smaller quantities of phenolic acids and flavonoids were present in the infusions than in the 75% ethanol extracts.

Only a few studies have focused on the assessment of phenolic acids and flavonoids present in roots, leaves, and fruits of species native to Asia and Russia. Data in the literature indicated that Kurkin et al. [30] identified free phenolic acids (syringic,* p*-coumaric, vanillic,* p*-hydroxybenzoic, caffeic, and ferulic acids) and depside (chlorogenic acid) in the roots of* E. senticosus* growing in Russia. Kurkin et al. [30] identified protocatechuic, chlorogenic, and caffeic acids in the roots of a Chinese sample. Bączek identified rosmarinic, chlorogenic, ferulic, and caffeic acids in the roots, fruits, and stem barks of six species [31, 32].

Protocatechuic acid has been identified in* Hibiscus sabdariffa* L. (2.8 and 11.9 mg/g aqueous and ethanol extracts from roselle calyx) and* Euterpe oleracea* Mart. (630 mg/L of oil) [33, 34]. In turn, the content of protocatechuic acid in* Allium cepa* L. was dependent on a type of raw material. The highest content was determined in a dried material (76.3 *μ*g/g) contrary to a fresh material (5.8 *μ*g/g) [35]. Comparing the results obtained in this work with the cited ones, it is concluded that* Eleutherococcus* spp. contain a higher amount of protocatechuic acid. This acid also occurs in various fruits such as berries (raspberry, blueberry, mulberry, cranberry, and gooseberry), wine, honey, and soybean. Protocatechuic acid has been found to have various activities such as antibacterial, antidiabetic, antiulcer, anti-inflammatory, and cardiac effects [36].

Data related to flavonoids in* Eleutherococcus* spp. are scarce and mainly relate to Załuski et al.'s previous studies [9, 26]. Zhao et al. [37] reported on the presence of hyperin in the green and mature fruits of* E. sessiliflorus* (630.7 and 430.1 *μ*g/g). Lee at al. [38] have detected rutin, hyperin, quercetin, and kaempferol in* E. divaricatus*, planted in Korea. Other studies on the content of rutin, hyperin, quercetin, and kaempferol, in different species of* E. divaricatus* and* E. sessiliflorus* collected in South Korea, have shown the differences dependent on the plant part. The roots and stems were rich in rutin (1.9, 3.5 and 4.2, 0.8 mg/g for* E. divaricatus* and* E. sessiliflorus*) [39].

## 4. Conclusions

Our results demonstrate that the fruits of* Eleutherococcus* species, rich in polyphenols and nutrients, have promising potential as a new income source of agriculture and industry in natural products and foods. The fruits may become ingredients of herbal teas or natural products where a high amount of phytochemicals and nutrients is needed.

## Supplementary Material

Detailed LC-ESI-MS/MS methods parameters are given in Supplementary Material.

## Figures and Tables

**Table 1:** Proximate composition (%) of the fruits of *E. divaricatus* and *E. sessiliflorus*^*∗*^.

Nutrient [%]	*E. divaricatus*	*E. sessiliflorus*
Moisture	7.72	8.21
Ash	5.53	4.89
Protein	16.70 ± 0.53	12.28 ± 0.39
Fat	6.9	3.26
Carbohydrates	2.5	25.7
Fibres	61.41 ± 20.26	45.63 ± 15.06
Calorific value	1046 kJ/100 g	1132 kJ/100 g
	255 kcal/100 g	273 kcal/100 g

^*∗*^Results are means ± standard deviation of triplicates.

**Table 2:** Mineral compositions of the fruits [mg/kg].

	Fe	Ca	Mg	K	Na	Cu	Zn	Mn	Se
*E. divaricatus*	30 ± 3.5	3310	1460	2100	1	5.3 ± 0.8	27.5 ± 2.8	105.5	0.84
*E. sessiliflorus*	20 ± 2.9	1730	1240	2790	25	3 ± 0.62	10 ± 1.8	4	0.41

**Table 3:** Fatty acid composition of the fruits of *E. divaricatus* and *E. sessiliflorus  *[%].

Fatty acid	*E. divaricatus* ^*∗*^	*E. sessiliflorus* ^*∗*^
C10:0	—	0.06
C12:0	0.06	0.15
C13:0	0.08	—
C14:0	0.16	0.19
C15:0	0.05	0.13
C16:0	3.76 ± 0.22	4.96 ± 0.29
C16:1	—	0.76
C17:0	0.06	0.10
C17:1	—	0.10
C18:0	0.85 ± 0.09	1.27 ± 0.14
C18:1n9c + C18:1n9t	**57.61** ± 1.86	**53.73** ± 1.74
C18:2n6c + C18:2n6t	**35.43** ± 0.43	**34.73** ± 0.42
C18:3n3 (alpha)	0.79 ± 0.06	2.27 ± 0.17
C20:0	0.19 ± 0.01	0.23 ± 0.01
C20:1	0.34	0.26
C22:0	0.35	0.63
C24:0	0.23	0.41
**SFA**	5.77	8.11
**MUFA**	57.95	54.84
**PUFA**	36.22	37.00
**Omega-3**	0.79	2.27
**Omega-6**	35.43	34.73

^*∗*^Results are means ± standard deviation of triplicates.

**Table 4:** TPC and TFC in extracts from the fruits of *E. divaricatus* and* E. sessiliflorus* (mg GAE/g and QEs/g dry extract^*∗*^).

Species	TPC	Flavonoid content		TFC
		FeCl_3_	DNPH	
^∧^ *E. divaricatus*	52.3 ± 0.5	12.3 ± 0.11	6.0 ± 0.04	18.4 ± 0.02
^∧^ *E. sessiliflorus*	45.3 ± 0.5	17.3 ± 0.08	5.7 ± 0.01	23.0 ± 0.03
^∙^ *E. divaricatus*	41.1 ± 0.5	12.0 ± 0.5	6.1 ± 0.5	18.2 ± 0.5
^∙^ *E. sessiliflorus*	40.6 ± 0.5	10.0 ± 0.5	5.9 ± 0.5	15.0 ± 0.5

^*∗*^Results are means ± standard deviation of triplicates.

^∧^The 75% ethanol extracts; ^∙^the infusions.

**Table 5:** Phenolic acid contents expressed in *µ*g per 100 g of dry weight of extracts.

	75% ethanol		Infusion	
	*E. divaricatus*	*E. sessiliflorus*	*E. divaricatus*	*E. sessiliflorus*
*Phenolic acids*				

Protocatechuic acid	693	818	270	267
Caffeic acid	133	0.8	22.8	19.8
*p*-Coumaric acid	Trace	Trace	5.2	5.3
Ferulic acid	11.1	6.1	4.1	5.9
Salicylic acid	0.15	Trace	Trace	Trace

*Flavonoids*				

Rutin	90	Trace	0.002	Trace
Hyperoside	780	360	120	280
Isoquercetin	Trace	100	Trace	Trace
Naringin	Trace	Trace	0.9	1.0
Astragalin	30.0	10	—	—
Naringenin 7-glucoside	5.0	3.0	—	4.0

—: not detected; Trace: trace amounts. Mean values of three replicate assays with standard deviation.

## Abbreviations

TPC: Total phenolics content
TFC: Total flavonoids content
MUFA: Monounsaturated fatty acids
PUFA: Polyunsaturated fatty acids
DNPH: 2,4-Dinitrophenylhydrazine
FeCl_3_: Aluminium chloride
GA: Gallic acid
HE: Hesperetin equivalent
QE: Quercetin equivalent
AAS: Atomic absorption spectroscopy.
