# Supplementary material for: Phytoconstituents and Nutritional Properties of the Fruits of Eleutherococcus divaricatus and Eleutherococcus sessiliflorus: A Study of Non-European Species Cultivated in Poland
Source: Oxid Med Cell Longev. 2017 Jan 15;2017:8374295. doi: 10.1155/2017/8374295 (PMC5274665; doi:10.1155/2017/8374295)
Supplement: Supplementary file 1 — Detailed LC-ESI-MS/MS methods parameters are given in Supplementary Material. [file 8374295.f1.docx]

**Supplementary Material**

**Phytoconstituents and nutritional properties of the fruits of *Eleutherococcus divaricatus* and *Eleutherococcus sessiliflorus* - a study of non-European species cultivated in Poland**

**Daniel Załuski ^1,*^ Marta Olech ^2^, Robert Verpoorte ^3^, Inamullah Khan ^4^, Rafał Kuźniewski ^1^, Renata Nowak ^2^**

*^1^ Department of Pharmacognosy, Ludwik Rydygier Collegium Medicum, Nicolaus Copernicus University, 9 Marie Curie-Skłodowska Street, 85-094 Bydgoszcz, Poland.*

*^2^ Chair and Department of Pharmaceutical Botany, Medical University of Lublin, 1 Chodźki Street, 20-093 Lublin, Poland*

*^3^ Natural Products Laboratory, Institute of Biology, Leiden University, 2300 RA Leiden, The Netherlands*

***^4^****Department of Pharmacy, University of Peshawar, 25120, Pakistan*

^*^Corresponding Author:

Daniel Załuski E-mail address: [daniel_zaluski@onet.eu](mailto:daniel_zaluski@onet.eu) Phone: +48 12 620-55-60.

***Keywords***: *Eleutherococcus*, pharma-nutrients, vegetarianism, minerals, GC/MS/FID fatty acids, LC-ESI-MS/MS polyphenols, flavonoids

Abstract The *Eleutherococcus* fruits have been consumed in Russia and Asia throughout the centuries. Currently, there is an increasing interest in these products by the community of Western Europe. Many people suffer from micronutrient deficiencies, known as malnutrition, what consequently influences on body condition. The aim of this study was to investigate pharmaconutrition proximate, mineral contents, fatty acid composition, Total Phenolics Content, Total Flavonoids Content, phenolics and flavonoids of the *Eleutherococcus divaricatus* and *Eleutherococcus sessiliflorus* fruits cultivated in Poland. *Eleutherococcus divaricatus* and *E. sessiliflorus* contain a high amount of protein and fibres (16.70% and 12.28%; 61.41% and 45.63%, respectively). The fruits were generally high in K (21 g/kg) and low in sodium (0.001 g/kg). In terms of fatty acid  composition, both species had a high amount of MonoUnsaturated Fatty Acids  (54.84-57.95%) and PolyUnsaturated Fatty Acids  (36.22-37.0%). Using LC-ESI-MS/MS, protocatechuic acid has been identified as the most abundant compound, ranging from 260 to 810 μg/100 g DE. Among flavonoids, hyperoside was found to be in the highest amount (120-780 μg/100 g DE). Considering a rich chemical composition of the fruits, a better understanding of their health benefits is important in order to increase their utility and to enrich dietary sources of health promoting compounds. Because of a high amount of protein and a low calorific value, the fruits should be considered food for vegans or vegetarians.

**TABLES**

**Table S1.** **LC-ESI-MS/MS analytical results of phenolic acids, including retention times, mass-to-charge ratio (m/z) and fragments obtained with given collision energy. Compounds confirmed by comparison with authentic standards.**

| Compound | Peak no. | T_R_  (min) | [M-H]^-^ | Products of [M-H]^-^ | Colision energy (eV) |
| --- | --- | --- | --- | --- | --- |
| Gallic acid | 1 | 0.74 | 168.7 | 124.9 | - 14 |
|  |  |  |  | 78.9 | - 36 |
| Protocatechuic acid | 2 | 1.70 | 152.9 | 107.8 | - 38 |
|  |  |  |  | 80.9 | - 26 |
| Gentisic acid | 3 | 2.70 | 152.8 | 107.9 | - 36 |
|  |  |  |  | 81 | - 30 |
| 4-OH-benzoic acid | 4 | 3.26 | 136.8 | 92.9 | - 18 |
| Vanillic acid | 5 | 4.49 | 166.8 | 107.9 | - 18 |
|  |  |  |  | 123 | - 12 |
| Caffeic acid | 6 | 4.65 | 178.7 | 134.9 | - 16 |
|  |  |  |  | 88.9 | - 46 |
| Syringic acid | 7 | 5.26 | 196.9 | 181.9 | - 12 |
|  |  |  |  | 122.8 | - 24 |
| p-Coumaric acid | 8 | 5.60 | 162.7 | 119 | - 14 |
|  |  |  |  | 93 | - 44 |
| Ferulic acid | 9 | 5.77 | 192.8 | 177.9 | - 12 |
|  |  |  |  | 133.9 | - 16 |
| Salicylic acid | 10 | 5.80 | 136.8 | 93 | - 16 |
|  |  |  |  | 75 | - 48 |
| Veratric acid | 11 | 5.80 | 180.7 | 136.9 | - 12 |
|  |  |  |  | 121.9 | - 18 |
| Synapic acid | 12 | 5.81 | 222.8 | 148.9 | - 20 |
|  |  |  |  | 121 | - 36 |
| 3-OH-cinnamic acid | 13 | 5.82 | 162.8 | 119 | - 14 |
|  |  |  |  | 91 | - 36 |
| Rosmarinic acid | 14 | 5.97 | 358.7 | 160.8 | - 20 |
|  |  |  |  | 132.6 | - 44 |
|  |  |  |  |  |  |

Compounds confirmed by comparison with authentic standards.

**Table S2. LC–MS/MS data for the calibration curves and LODs, LOQs values for each analyzed phenolic acid.**

| **Compound** | **LOQ**  **[ng μl^-1^]** | **LOD**  **[ng μl^-1^]** | ***r^2^*** | **Linearity range**  **[ng μl^-1^]** |
| --- | --- | --- | --- | --- |
| Gallic acid | 0.10 | 0.05 | 0.9994 | 0.10 – 10.00 |
| Protocatechuic acid | 0.02 | 0.01 | 0.9991 | 0.025 – 3.13 |
| Gentisic acid | 0.015 | 0.008 | 0.9993 | 0.025 – 25.00 |
| 4-OH-benzoic acid | 0.10 | 0.05 | 0.9971 | 0.10 - 2.50 |
| Vanillic acid | 0.20 | 0.10 | 0.9999 | 0.2 - 50 |
| Caffeic acid | 0.08 | 0.04 | 0.9972 | 0.08 - 1.25 |
| Syringic acid | 0.10 | 0.05 | 0.9997 | 0.1 – 50.00 |
| p-Coumaric acid | 0.061 | 0.018 | 0.9971 | 0.10 – 10.20 |
| Ferulic acid | 0.025 | 0.01 | 0.9997 | 0.025 – 5.00 |
| Salicylic acid | 0.02 | 0.01 | 0.9986 | 0.02 - 0.50 |
| Veratric acid | 0.70 | 0.40 | 0.9977 | 0.50 – 25.00 |
| Synapic acid | 0.025 | 0.007 | 0.9987 | 0.025 – 5.00 |
| 3-OH-cinnamic acid | 0.05 | 0.02 | 0.9994 | 0.05 - 2.50 |
| Rosmarinic acid | 0.01 | 0.005 | 0.9985 | 0.025 – 25.00 |

| Analyte^a^ | Peak no. | T_R_  (min) | [M-H]^-^ | Products of [M-H]^-^ | Colision energy (eV) |
| --- | --- | --- | --- | --- | --- |
| Luteolin 3,7-diglucoside | 1 | 6.25 | 609.1 | 285.0 | -50 |
|  |  |  |  | 447.0 | -32 |
| Rutin  (Quercetin 3-*O*-rutinoside) | 2 | 8.10 | 608.7 | 299.6 | -46 |
|  |  |  |  | 270.9 | -60 |
| Hyperoside  (Quercetin 3-*O*-galactoside) | 3 | 9.30 | 462.7 | 299.7 | -28 |
|  |  |  |  | 254.7 | -42 |
| Luteolin 7- *O*-glucoside | 4 | 9.35 | 446.8 | 284.8 | -30 |
|  |  |  |  | 132.9 | -78 |
| Isoquercetin  (Quercetin 3-*O*-glucoside) | 5 | 9.52 | 462.7 | 299.7 | -30 |
|  |  |  |  | 270.7 | -44 |
| Kaempferol 3-*O*-rutinoside | 6 | 9.90 | 592.7 | 284.8 | -38 |
|  |  |  |  | 226.7 | -68 |
| Naringin  (Naringenin 7-*O*-rhamnosidoglucoside) | 7 | 11.70 | 579.1 | 151.0 | -54 |
|  |  |  |  | 271.0 | -42 |
| Astragalin  (Kaempferol 3-*O*-glucoside) | 8 | 11.90 | 446.7 | 254.8 | -40 |
|  |  |  |  | 226.8 | -54 |
| Quercitrin  (Quercetin 3-*O*-rhamnoside) | 9 | 12.45 | 446.8 | 299.7 | -30 |
|  |  |  |  | 270.7 | -40 |
| Apigenin 7-*O*-glucoside | 10 | 12.70 | 430.7 | 267.7 | -38 |
|  |  |  |  | 116.9 | -84 |
| Luteolin 4’-*O*-glucoside | 11 | 12.90 | 447.0 | 285.1 | -28 |
|  |  |  |  | 133.0 | -74 |
| Naringenin 7-*O*-glucoside | 12 | 13.20 | 432.7 | 270.8 | -22 |
|  |  |  |  | 118.9 | -64 |

**Table S3.**  **LC-ESI-MS/MS analytical results of flavonoids, including retention times, mass-to-charge ratio (m/z) and fragments obtained with given collision energy. Compounds confirmed by comparison with authentic standards.**

**Table S4. LC–MS/MS data for the calibration curves and LODs, LOQs values for each analyzed flavonoid.**

| **Compound** | **Calibration curve** | ***r^2^*** | **LOD**  **[ng mL^1^]** | **LOQ**  **[ng mL^-1^]** | **Linearity range**  **[ng mL^-1^]** |
| --- | --- | --- | --- | --- | --- |
| Luteolin 3,7-diglucoside | y = 656 x + 1.85e+003 | 0.9999 | 0.02 | 0.05 | 0.5 - 2500 |
| Rutin | y = 245x - 309 | 0.9999 | 2.5 | 5 | 5 - 5000 |
| Hyperoside | y = 295 x + 6.52e+003 | 0.9988 | 10 | 20 | 20 - 2500 |
| Luteolin 7-glucoside | y = 701 x + 6.65e+003 | 0.9998 | 2.5 | 5 | 5 -2500 |
| Isoquercetin | y = 308 x + 5.35e+003 | 0.9993 | 10 | 20 | 20 - 2500 |
| Kaempferol 3-rutinoside | y = 266 x + 127 | 0.9999 | 5 | 10 | 10 - 5000 |
| Naringin | y = 730 x - 192 | 0.9999 | 2 | 5 | 5 - 2500 |
| Astragalin | y = 385 x + 1.58e+003 | 0.9999 | 3 | 7.5 | 10 - 2500 |
| Quercetrin | y = 453 x + 5.05e+003 | 0.9998 | 5 | 10 | 10 - 5000 |
| Apigenin 7-glucoside | y = 902 x + 4.77e+003 | 0.9996 | 2 | 4 | 5 - 1000 |
| Luteolin 4-glucoside | y = 1.32e+003 x + 2e+004 | 0.9996 | 1 | 2.5 | 8 - 2500 |
| Naringenin 7-glucoside | y = 402 x + 7.72e+003 | 0.9996 | 0.01 | 0.04 | 0.1 - 1000 |
